# Therapeutic Potential of Fulvic Acid in Chronic Inflammatory Diseases and Diabetes

**DOI:** 10.1155/2018/5391014

**Published:** 2018-09-10

**Authors:** John Winkler, Sanjoy Ghosh

**Affiliations:** Department of Biology, IKBSAS, University of British Columbia-Okanagan, Canada

## Abstract

Chronic inflammatory diseases like diabetes are on a rise in the Western world. Based on the tsunami of new cases every year, new therapeutic measures must be considered. A promising avenue might involve the attenuation of underlying inflammation through natural health products (NHPs). This is because most NHPs have a rich history in traditional medicine and might be considered safer under appropriate doses and conditions. However, the biggest impediment in NHP research is that rarely do these products come with verified health benefits or dosing schedules established through modern scientific research. Fulvic acid (FvA), one such NHP, comes from humic substances produced by microorganisms in soil. Traditional medicine and modern research claim FvA can modulate the immune system, influence the oxidative state of cells, and improve gastrointestinal function; all of which are hallmarks of diabetes. This minireview outlines the available peer-reviewed research on FvA and examines its anecdotal health claims. We show that although available research has been minimal, there is substantial evidence to pursue FvA research in preventing chronic inflammatory diseases, including diabetes.

## 1. Introduction

Diseases associated with chronic inflammation such as diabetes, cardiovascular disease, and colitis have been increasing. For example, the number of people living with diabetes in Canada in 2015 was 3.4 million and is predicted to reach 5 million by 2025 [1]. Millions of dollars have been poured into the development of drugs to treat these diseases with little success [2]. Thus, it is time to explore new avenues in treating and preventing chronic inflammatory diseases. Natural health products (NHPs) may provide a promising route in this quest for alternatives. First, they require little to no development, and second, they are often accompanied by a history rich in traditional medicine [3]. Fulvic acid (FvA) is a publically available NHP that combines those two facts and may provide promising outcomes for chronic inflammatory diseases.

**Figure 1:**
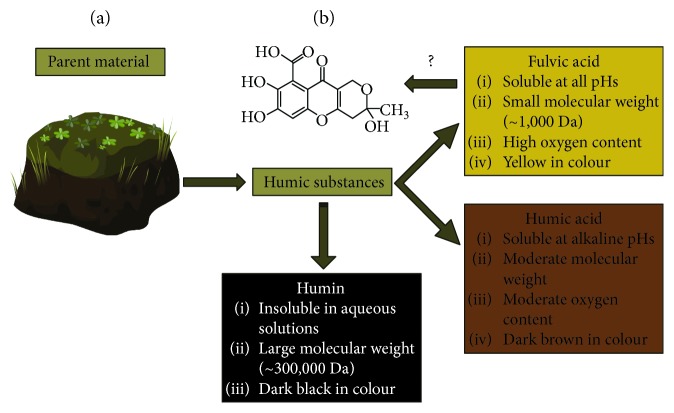
The characterization and classification of humic substances. Adapted from [5]. (a) Humic substances are isolated from various parent material like peat, coal, water, and soil through a series of precipitation/dissolution steps; their general characteristics are highlighted in each box [9, 10]. (b) Proposed composition of fulvic acid by Health Canada.

FvA is a subclass of diverse compounds known as humic substances, which are by-products of organic degradation from microorganisms [4]. What separates FvA from other humic substances (HS) is a set of physical and chemical properties shown in Figure 1(a), indicated by Stevenson [5] and followed by the International Humic Substance Society (IHSS; St. Paul, Minnesota, USA) [4, 6]. By definition, FvAs consist in small molecular weight, hydrophilic, carboxylic-containing molecules. The other HS have higher molecular weight and different solubility and oxygen content. The structure of FvA has been proposed by many authors to be a mixture of covalently linked phenolic, quinoid, and benzene carboxylic acid compounds [6]. It is important to note that FvA can change with geographic location. The parent material from which FvA originates influences oxygen, nitrogen, aromatic ring, and carbon content [7]. For example, in Israel, FvA isolated from clay contains ~2.0% (m/m) nitrogen, and FvA isolated from sand contains ~4.4% (m/m) nitrogen [7]. In addition, FvA isolated from Israel has ~49% (m/m) carbon whereas FvA from Italy has ~39% carbon content [7]. Health Canada indicates that FvA is consistent with the structure shown in Figure 1(b) [8].

FvA has been indirectly utilized in traditional Indian medicine (“Ayurveda”) for roughly 3000 years [3]. The substance called Shilajit, a tar-like exudate from the Himalayas, contains about 15–20% FvA and is used for medicinal purposes. As per ancient texts, Shilajit can have immune-modulation, antioxidant, diuretic, antihypertensive, and hypoglycaemic effects [3]. In addition, when applied externally, it is claimed to be an antiseptic and analgesic [11]. Reviews on Shilajit indicate that the intake is safe; however, the pharmacological dosing of such molecules remain unknown [3]. Despite such lack of information, Shilajit/FvA is currently available as a nutraceutical to the public [12]. The purpose of this review is to investigate and highlight the current knowledge base regarding FvA and its effects on animals and animal cells.

## 2. Immunomodulation by FvA

**Figure 2:**
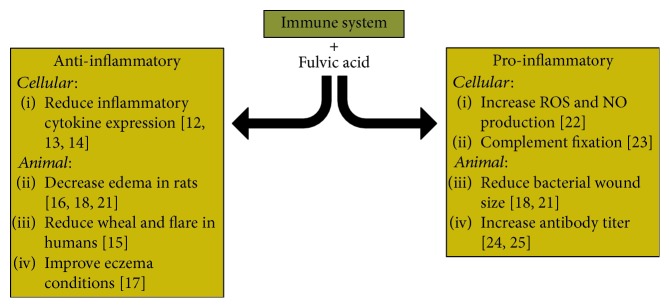
Known literature for the effects of fulvic acid on the immune system. Fulvic acid is shown to induce as well as reduce inflammation.

The most adequately researched claim of FvA is its ability to modulate the immune system. However, the outcomes of such studies remain controversial. FvA can be both proinflammatory and anti-inflammatory in animal systems. The available literature regarding the effects of FvA on the immune system is summarized in Figure 2.

### 2.1. Anti-inflammatory Effects of FvA

Asthma, allergies, and eczema, along with many other disorders, can be associated with overactive immune cells [13]. In these cases, anti-inflammatory drugs are critical for reducing symptoms. Several studies indicate that FvA can act as an anti-inflammatory by reducing the release of proinflammatory mediators from cells. First, Junek et al. show that FvA at 200 *μ*g/mL can reduce tumour necrosis factor alpha (TNF-*α*) expression after exposure to the endotoxin Lipopolysaccharide (LPS) in differentiated human monocytes (U937) [14]. FvA also is shown to reduce cyclooxygenase 2 (COX2) and prostaglandin E2 (PGE2) secretion after homocysteine stimulation in primary human monocytes [15]. FvA from solubilized sludge (SS-FA) is shown to reduce B-hexosaminidase and histamine release in immunoglobulin-E-sensitized mast cells and basophil cells [16]. This information suggests that FvA can have anti-inflammatory and antiallergy effects. Yamada et al. also show SS-FA decreases TNF-*α*, interleukin-4 (IL-4), and IL-13 from mast cells.

Unfortunately, *in vivo* studies into the effects of FvA have been too few and sporadic. A pilot clinical study shows coal-derived FvA (oxifulvic acid) at 4.5% (w/w) reduces wheal and flare size after allergen challenge in humans [17]. The reduction by FvA shows similar results to 1% hydrocortisone. The anti-inflammatory properties of oxifulvic acid are also shown in mice [18]. In the study, mice sensitized with dinitrofluorobenzene on the ear and then again challenged 6 days later see a reduction in swelling with FvA treatment comparable to steroid medication. A patented isolation procedure yielding carbohydrate-derived fulvic acid (CHD-FvA) mimics the above information almost entirely. In a randomized clinical trial, topical administration of CHD-FvA is shown to significantly reduce eczema rash in humans [19]. However, a burning sensation was also reported in this study. In addition, oral ingestion of CHD-FvA isolated from South Africa at 100 mg/kg can reduce paw edema in rats at levels similar to nonsteroidal anti-inflammatory drugs [20]. Overall, the above studies promise the potential of FvA to treat overactive immune disorders, specifically eczema.

### 2.2. Proinflammatory Effects of FvA

The immune system is an integral part of human health and has evolved to be a complex organization in which we rely. It provides protection against pathogens and stops tumour growth by initiating the inflammatory response [21, 22]. Interestingly, FvA has been shown to enhance inflammation in animals too. In the same study by Sabi et al. [20], topically applied CHD-FvA can reduce the size of wounds infected with *Staphylococcus aureus*, thus stopping the progression of the infection. CHD-FvA is also suggested to reduce the size of wounds infected with antibiotic-resistant pathogens [23]. This suggests a bimodal effect of FvA that not only suppresses immune function but also stimulates it. Essentially, it provides evidence that FvA ensures proper immune function. Schepetkin et al. show that FvA can activate isolated murine macrophages from the peritoneal cavity. Nitric oxide (NO) and reactive oxygen species (ROS) important for killing bacteria and intracellular signalling increase in FvA-treated peritoneal macrophages [24]. They do however indicate cell viability is reduced with 100 *μ*g/mL of FvA, questioning studies by Junek et al. [14]. In addition, Schepetkin et al. also show that IHSS FvA standards and low molecular-weight extracts from Nepal Shilajit can fix the complement system [25]. This gives rise to the thought that FvA can activate the immune system when needed to protect against infection and foreign pathogens.

### 2.3. Detrimental Immune Effects of FvA

Several studies indicate that FvA may be harmful too. FvA derived from Hungarian lignite can activate humoral immunity and reduce thyroid function in rats [26]. In this study, FvA increases antibody titre against ovalbumin 14 days and 26 days after challenge. They also indicate that lymphocyte diameter increases in rats, a sign of cellular activation [26]. These results are not isolated; Kunavue and Lien show increases in IgG antibodies in weanling pigs after FvA treatment [27]. No significant increase is found in lymphocyte, monocyte, or granulocyte numbers in the blood however. Unfortunately, Kunavue and Lien do not mention the location and isolation procedure of the FvA used.

Such discordant effects seem to result from variances in therapeutic dosages and/or the origin of FvA in the study. Thereby, it is an absolute must to establish safe dosing for FvA depending on its source in order to treat/prevent immune-modulatory disorders.

## 3. Oxidative Stress

**Figure 3:**
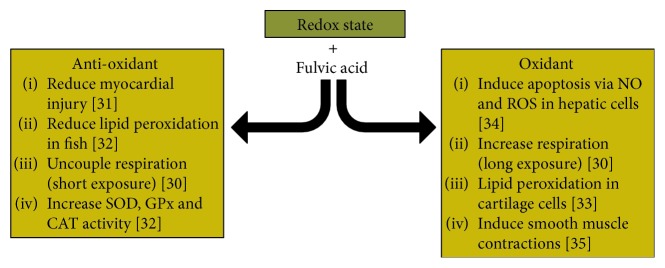
Known literature for the effects of fulvic acid on the redox state of cells. Fulvic acid is shown to have various effects which include increasing oxidative stress but also reducing it.

Oxidative stress is closely linked to chronic inflammatory diseases [28]. Oxidative stress is described as an imbalance of highly reactive oxygen species (ROS) compared to antioxidants [29]. When the cellular equilibrium shifts towards higher ROS, endogenous antioxidants like glutathione (GSH) and superoxide dismutase (SOD) are outmatched. This leads to cellular dysfunction, lipid peroxidation, and possible cell death [30]. The effects of FvA on the oxidative state in cells and animals are summarized in Figure 3.

### 3.1. Antioxidant Capabilities of FvA

FvA has been shown to sequester superoxide radicals and other ROS outside of the cell [31]. Inside the cell though, FvA can uncouple the electron transport chain in liver mitochondria, which is associated with lowering ROS production [32]. In addition, the most promising *in vivo* study regarding the antioxidant ability of FvA is the reduction of oxidative stress markers after isoproterenol (ISO) induced myocardial damage in rats. Shikalgar and Naikwade show that FvA at 300 mg/kg/day for 4 weeks decreases lipid peroxidation and myocardial damage markers after ISO and significantly increases GSH, SOD, and catalase (CAT) levels [33]. Another study confirms this information in fish. After feeding FvA for 60 days, a decrease in lipid peroxidation and an increase in the expression of SOD, CAT, and glutathione peroxidase (GPx) are seen [34].

### 3.2. Oxidant Capabilities of FvA

Just like in inflammation, FvA can also cause oxidative damage instead of preventing it. FvA increases oxidative stress when exposed to isolated cartilage cells from 12-day-old embryonic chicks [35]. This study contrasts ones showing a decrease in lipid peroxidation and further suggests FvA as a causative factor in Kashin-Beck disease. An underlying factor for elevated oxidative stress is that FvA can increase cellular respiration rates at extended exposure in rat mitochondria, which may lead to the production of more oxygen radicals [30]. FvA has been shown to increase oxidative markers like hydrogen peroxide and nitric oxide and induce apoptosis in hepatic cancer cell lines [36]. Similarly, FvA can increase smooth muscle contractions, which can be linked to oxidative damage [37].

## 4. Gut Health

**Figure 4:**
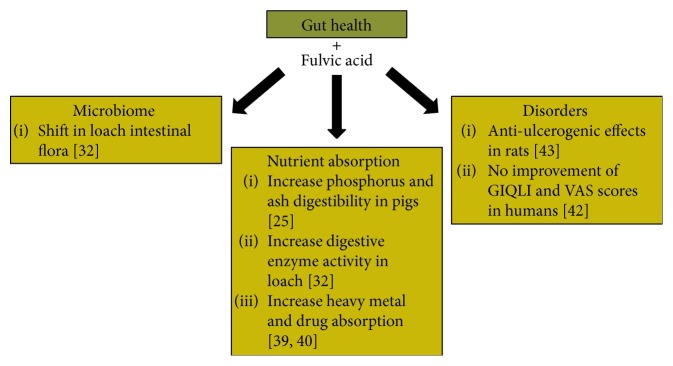
Known literature for the effects of fulvic acid on gut health. Fulvic acid has been shown to influence the microbiome, nutrient absorption, and gut disorders.

The gut forms the interphase of the outside world, the microbiome, and the host. Sufficient evidence shows that poor gut health can lead to inflammation and disease [38]. In agriculture, FvA has been shown to influence the soil microbe composition and be able to conjugate itself to various minerals, aiding in the uptake in plants [39, 40]. As a result, FvA is suggested to improve the gut flora, nutrient absorption, and heal adverse disorders related to the gut. Below is the available content regarding FvA effects on gut health and summarized in Figure 4.

### 4.1. Shifting the Microbiota

In regard to the microbiota, very little information is available in animals. A study by Gao et al. show that FvA at 1.5% (w/w) can modulate the gut microflora in loach (*Paramisgurnus dabryanus*) fish [34]. After 60 days of feeding, the abundance of Proteobacteria phyla decreases and Firmicute levels increase in the intestine. In addition, 10 bacteria genera were influenced by FvA treatment. Bacteria of note include an increase in *Variovorax*, *Lactococcus*, and *Lactobacillus* and a decrease in *Serratia* and *Acinetobacter*. This is the only study investigating the effect of FvA on the microbiome.

### 4.2. Enhancing Nutrient Absorption

Gao et al. [34] also show FvA increases the activity of digestive enzymes like lysozyme, proteases, and acid/alkaline phosphatases in fish. This is strengthened in part by an intensive study investigating nutrient digestibility in pigs [27]. FvA supplemented at 200 ppm in fed improved phosphorus and ash digestion but interestingly had no effect on fat and protein digestibility, contrasting data found in loach.

FvA is shown to influence the bioavailability of heavy metals in animal models as well. FvA can increase the absorption of copper in porcine oviductal epithelial cells and simultaneously reduces its toxicity [41]. In addition to nutrients, FvA has been shown to mediate drug delivery in rats too [42]. Carbamazepine (CBZ) a common anticonvulsant has a low bioavailability, but when conjugated to FvA, absorption across an everted rat intestinal sac increases along with concentrations of CBZ in the blood plasma. FvA has been shown to increase absorption of nutrients and drugs; thus, a concern is the absorption of pollutants and toxins into the blood. However, in a study by Qiang et al., FvA does not increase the absorption of perfluorooctanesulfonate (PFOS) in carp, a conclusion based on the amount of PFOS in the feces of fish compared to controls [43].

### 4.3. Improve Gut Disorders

A preliminary clinical study exists which investigates the efficacy of probiotics in combination with FvA in gastrointestinal (GI) disorders [44]. Unfortunately though, all groups including ones with FvA see no improvement in Gastrointestinal Quality of Life Index (GIQLI) and Visual Analogue Scale (VAS) scores for GI symptoms. This study does showcase the safety of FvA intake over a 12-week period. Even though FvA is not shown to have an effect in the previous study, FvA isolated from Shilajit shows promise to be antiulcerogenic during several battery tests in albino rats [45].

## 5. Potential of FvA in Diabetes

Type 2 Diabetes Mellitus (T2DM) is characterized by improper insulin signalling and attenuated glucose uptake into cells [46]. This can lead to prolonged hyperglycaemia after feeding and adverse symptoms [46]. The cause of diabetes remains a mystery, but research associates inflammation, oxidative stress, and changes in the gut microbiome among the many causative factors [47]. Shilajit, which contains FvA, has been shown to reduce hyperglycaemia in diabetic rats and increase SOD activity in pancreatic beta cells [48, 49]. Unfortunately though, there is no direct evidence in the English language showing only FvA in preventing T2DM symptoms. However, the accumulative effects highlighted in this review and the last two studies suggest its therapeutic potential.

Those with T2DM show signs of chronic inflammation and elevated proinflammatory cytokines in serum like TNF-*α*, IL-1, and IL-6 [47]. FvA is shown to reduce these types of cytokines and proinflammatory markers in animal models [14, 16]. In addition, a proposed treatment regime for T2DM involves nonsteroidal anti-inflammatory drugs (NSAIDs) to alleviate symptoms [50]. FvA might fit as an adjunct treatment to reduce markers of oxidative stress and inflammation as FvA can act in a similar manner to NSAIDs [20]. FvA might also reduce oxidative damage and increase antioxidant enzymes like SOD, CAT, and GPx [33]. Beta cells, which are responsible for insulin production, undergo oxidative damage during T2DM [51]. Protecting the redox state of beta cells may prove beneficial in preventing T2DM. Lastly, patients with T2DM are found with a change in gut microbial composition, and FvA may influence the bacterial community [34].

## 6. Conclusions

The information gathered in this review indicate that FvA can act as an immune modulator, influence the redox state, and potentially affect gut health. FvA is shown to decrease proinflammatory markers but also activate the immune system to kill bacteria. It is shown to reduce oxidative stress and even induce apoptosis in hepatic cancer lines. FvA is shown to also influence the microbiome and possibly improve gut function. FvA appears to have a yin-yang effect when it comes to these physiological states. This trend can be seen with most drugs and NHPs; however, toxicity may manifest itself at high intake and poor administration [52, 53].

Although the supporting literature is minimal, if considered in combination, the potential for FvA to be a candidate in preventing inflammatory diseases like diabetes arises. This is promising as our current approach to these kinds of diseases is lacking. It is important to note that FvA research in some cases is conflicting, which is thought to be a result of variance in dosage, parent material, and isolation procedure. In addition, there is no consensus on the structure of FvA, a standard isolation, or parent material. Thus, it is of paramount concern to reconcile these factors and establish dosing for age groups and differing FvA. This will help make conclusive statements regarding FvA function and its influence on immune-related diseases.
